# Dynamic Perviousness: A Novel Imaging Marker for Predicting Mechanical Thrombectomy Outcomes in Acute Ischemic Stroke

**DOI:** 10.3390/diagnostics14111197

**Published:** 2024-06-06

**Authors:** Daniel F. Toth, Gergely Bertalan, Priska Heinz, Jawid Madjidyar, Patrick Thurner, Tilman Schubert, Zsolt Kulcsar

**Affiliations:** 1 Department of Neuroradiology, Clinical Neuroscience Center, University Hospital Zürich, 8091 Zürich, Switzerlandpatrick.thurner@usz.ch (P.T.);; 2 Department of Biostatistics, Epidemiology, Biostatistics and Prevention Institute, University of Zürich, 8001 Zürich, Switzerland

**Keywords:** acute ischemic stroke, mechanical thrombectomy, dynamic perviousness of thrombi, computed tomography

## Abstract

Background: The predictive value of thrombus standard perviousness (SP) in acute ischemic stroke (AIS) for the technical success rates of mechanical thrombectomy (MT) or functional outcomes is not yet conclusive. We investigated the relationship between dynamic perviousness (DP) and revascularization results using time-dependent enhancement curve types determined with computed tomography (CT). Methods: A retrospective analysis of 137 AIS patients was performed. DP was calculated as the thrombus attenuation increase (TAI) using three time points and categorized into four groups: (1) no enhancement (CNE); (2) late enhancement (CLE); (3) early enhancement with washout (CW); (4) early enhancement without washout (CNW). Associations with the technical success rate and functional outcomes were assessed. Results: Late enhancement (CLE) had approximately two times higher odds for successful MT as compared to clots with other enhancement dynamics. The odds ratios (logistic regression model with CNW as the reference) for the TICI III scores were 4.04 (*p* = 0.067), 1.82 (*p* = 0.3), and 1.69 (*p* = 0.4) for CLE, CW, and CNE, respectively. The NIHSS scores at discharge and mRS scores at three months showed regression coefficients (linear regression model with CNW as reference) of −3.05 (*p* = 0.10), −1.17 (*p* = 0.51), and −1.24 (*p* = 0.47); and −1.30 (*p* = 0.097), −0.85 (*p* = 0.25), and −0.15 (*p* = 0.83) for CLE, CW, and CNE, respectively. Conclusions: Thrombi with late enhancement patterns showed a higher revascularization rate and better outcomes as compared to clots with early uptake or no washout.

## 1. Introduction

Mechanical thrombectomy (MT) became the gold standard treatment of acute ischemic stroke (AIS) due to the large vessel occlusion. The goal of MT is fast and complete revascularization, which is associated with a good clinical outcome and lower mortality rate [1,2]. Recently, efforts have been made to predict the thrombus properties from pre-treatment computed tomography (CT) imaging, as such predictions could lead to an optimized revascularization strategy and increased functional outcome.

Thrombus perviousness is an imaging biomarker that describes the contrast material’s penetration into the clot. Using pre-interventional CT imaging, standard perviousness (SP) is described by the thrombus attenuation increase (TAI) measured as the mean clot density difference based on non-contrast CT (NCCT) and CT angiography (CTA). It is believed that thrombus perviousness is closely linked to the physical structure of the clot, which in turn influences the technical success rate of the MT and functional outcome. Perviousness is thought to be a biomarker of elasticity, which may be associated with less fragmentation. Several studies have reported that thrombus perviousness is associated with better recanalization and clinical outcomes [1,2,3,4,5,6]. However, contradictory studies have also been reported, in which no correlations between SP and MT success rates or clinical outcomes were found [7,8,9,10].

The conflicting results for SP may be partly related to the short time between the contrast material’s administration and the acquisition of the CTA results, not permitting sufficient time for the contrast agent to interact with the occlusive thrombus [11]. In addition, SP measurements based on single-phase CTA and using only two time points for TAI calculations do not take into account the late uptake and early washout of the contrast agent, possibly resulting in imprecise clot characterization. Therefore, in this study, we used an additional late venous phase (CTV) time point after CTA and determined the late enhancement and early washout components of the contrast agent’s uptake, which were analyzed together with the standard perviousness for their predictive value regarding the MT results and functional outcome. We name this approach dynamic perviousness (DP) in this work.

## 2. Materials and Methods

### 2.1. Patients

**Figure 1:**
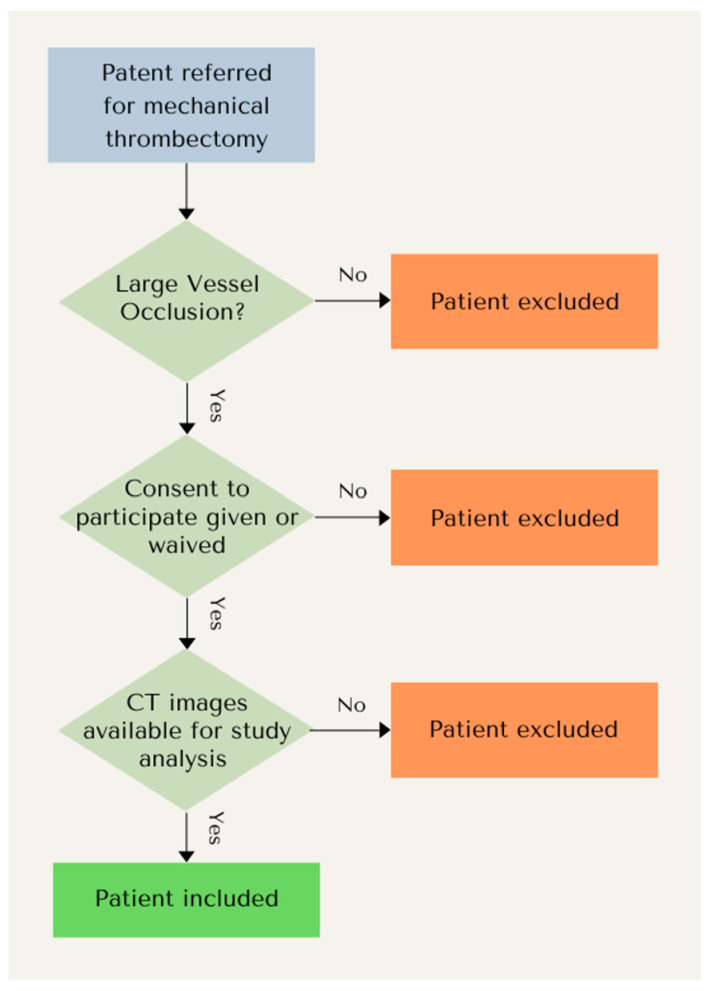
Flowchart of inclusion and exclusion criteria.

This study was approved by the regional ethics committee (BASEC Nr: 2022-00422). A retrospective analysis of the prospective database of 475 consecutive patients referred for mechanical thrombectomy due to LVO at our hospital between 2019 and 2021 was performed. All patients had a diagnosis of an anterior or posterior circulation LVO. We included patients with occlusions of the intracranial internal carotid artery (ICA), proximal middle cerebral artery (MCA) up to the proximal M2 segment, and basilar artery. The exclusion criteria were an unsuitable thrombus, either due to calcification or due to distal location, including any location at or distal to the distal M2, A1, or P1 segments of the MCA, anterior cerebral artery (ACA), and posterior cerebral artery (PCA), respectively, or an exclusively extracranial thrombus in the ICA (*n* = 84); the unavailability of CT imaging due to either an MRI being performed (*n* = 54) or imaging being performed at an external referring site with no permanent storage in our PACS (*n* = 81); or CT imaging being insufficient for the analysis due to a lack of a late or venous phase (*n* = 46), lack of an unenhanced series due to previous contrast administration for another study or procedure (*n* = 9), or strong motion artefacts or an insufficient imaging resolution of below 0.8 mm in voxel size (*n* = 43). Finally, a further set of eligible patients did not consent to participate and were not included (*n* = 21). This resulted in a final number of 137 patients. The inclusion and exclusion parameters are summarized in Figure 1.

For these patients, we collected standard demographic data, as well as data on the MT and functional outcomes. The number of MT passes and Thrombolysis In Cerebral Infarction (TICI) scale (ranging from 0 for no revascularization to 2c for near complete and 3 for full revascularization) and NIH Stroke Scale (NIHSS) scores, as well as the Modified Rankin Scale (mRS) scores at admission, discharge, and three-month follow-up, were included in the analysis.

The study protocol was approved by the regional ethical board and conformed to the ethical guidelines of the Declaration of Helsinki.

### 2.2. Imaging

The CT was performed on a range of scanners from different vendors, including Siemens Somatom X.cite, Somatom Definition Flash, Somatom Definition AS+, and Somatom Definition Edge Plus (Siemens, Erlangen, Germany) systems, as well as GE Revolution (General Electric, Boston, MA, USA) and Philips Brilliance iCT 256 (Philips, Amsterdam, The Netherlands) systems. The three-phase CT clinical protocol consisted of NCCT, an arterial phase measured with CTA, and a CTV after intravenous contrast agent injection. The CTV was timed with a mean delay of 70 ± 28 s after the CTA. A fixed tube voltage of 120 kV for both the NCCT and CTV was used. The tube voltage for the CTA depended on the scanner and patient characteristics, and was generally between 80 and 90 kV.

### 2.3. Image Analysis

**Figure 2:**
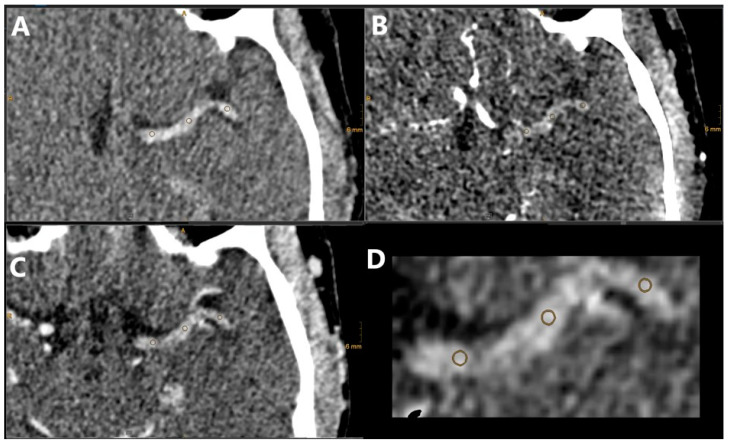
NCCT (**A**), CTA (**B**), and CTV (**C**) images of our clinical protocol for a patient with distal ICA and proximal M1 occlusions on the left side. The TAI was calculated between the NCCT and CTA, NCCT and CTV, and CTA and CTV results using spherically defined ROIs as illustrated in (**D**), overlaid on the NCCT image. The ROIs were defined manually by considering the clot area based on the NCCT and CTV results.

The analysis of the CT images was performed on our clinical PACS system (DeepUnity R20, Dedalus, Florence, Italy). The built-in 3D co-registration tool (3D Fit) was used to align the NCCT, CTA, and CTV series with manual corrections if necessary. A multiplanar reformat tool was used, where needed, for full visualization of the clot. The location of the clot was noted and the length was measured using a combination of hyperdensity for the NCCT and distal flow for the CTV. The TAI was measured similarly as by Santos et al. [4]. Three regions of interest (ROI) were placed over the length of the clot with a size of 1 mm each, and the density value in Hounsfield units (HU) was noted in the three imaging series simultaneously (Figure 2). This assessment was performed by an interventional radiologist with 10 years of experience (DT).

The three measurements per phase were averaged to a single density value for the NCCT, CTA, and CTV scans. The changes in density from the NCCT to CTA results, as well as from the NCCT to CTV and CTA to CTV results, were calculated. From this, we determined four patterns of thrombus perviousness: (1) no contrast enhancement (CNE; maximum increase of 5 HU over all phases); (2) late enhancement (CLE; maximum increase of 5 HU from NCCT to CTA and more than 5 HU from NCCT to CTV); (3) early enhancement with washout (CW; increase of more than 5 HU from NCCT to CTA and minimum decrease of 5 HU from CTA to CTV); (4) early enhancement without washout (CNW; increase of more than 5 HU from NCCT to CTA and maximum decrease of 5 HU from CTA to CTV).

### 2.4. Statistical Analysis

The statistical analysis was performed by the Department of Biostatistics at our Institution. Statistical reports were generated using R version 4.2.2 [12] and MATLAB 2022 (MathWorks, Inc., Natick, MA, USA). The association between the clot enhancement types and successful recanalization was assessed using logistic regression, once using a simple model and once by adjusting for clot length. Successful recanalization was defined as a TICI score of IIc or III, implying either total or near total reperfusion of the occluded vessel territory. The clinical outcomes, including the NIHSS score at discharge and functional outcome defined by the mRS at 3-month follow-up, were assessed by performing linear regression. Poisson regression was used to analyze whether the number of passes required to achieve the final result was associated with the uptake behavior.

The data are shown as means and standard deviations for normally distributed variables and medians and interquartile ranges (IQRs) for non-normally distributed variables, as well as numbers and percentages of the total for categorical variables. The statistical analysis results are presented as point estimates of mean differences (beta coefficients), odds ratios (OR) or rate ratios with two-sided 95% confidence intervals (CI) and two-sided *p*-values.

## 3. Results

### 3.1. Clinical Data

The demographics and clinical data are summarized in Table 1. The median (range) age was 75.5 years (17–94), with 81 males (59%) and 56 females (41%). Based on the NIHSS scores at admission, the majority of patients had moderate (NIHSS of 5–15, *n* = 56) or moderate to severe (NIHSS of 5–20 *n* = 41) stroke symptoms. The remaining patients presented with minor (NIHSS below 5, *n* = 14) or severe (NIHSS above 20, *n* = 25) symptoms. The median NIHSS was 15 (IQR 9–18) and 10 patients presented with a coma. The intervention was considered successful (TICI 2c or 3) in 109 out of 137 patients (79.6%). The numbers of MT passes ranged from 1 to 9, with a median of 2 and 57 instances of successful first pass recanalization (41.2%). At the time of discharge assessment, 26 patients had not survived. The 111 remaining patients had NIHSS scores ranging from 0 to 22 with a median of 2 (IQR 1–9), with 22 having no residual symptoms (18.3%). At the 3-month follow-up, mRS data were not available for 35 patients. Out of the remaining 76 patients, 44 achieved good functional outcomes (mRS 0–2, 43.7%).

### 3.2. Imaging and Perviousness Data

The imaging data are summarized in Table 2. The primary location of the clot was in the intracranial ICA (including long or tandem occlusions reaching the ACA or MCA) in 43 cases, the M1 segment of the MCA in 69 cases, the proximal M2 segment of the MCA in 19 cases, and the basilar artery (BA) in 6 cases. The average clot length was 18 mm, with a range of 3 to 61 mm (SD = 10 mm). The mean density of the clot was 64 HU (±9.6) based on the unenhanced images. There were no significant differences between the enhancement groups. The mean density based on the arterial images was 74 HU (±22), with the values ranging from 61 ± 8 in the late enhancement (C_LE_) group to 95 ± 31 in the early enhancement with washout (C_W_) group. In the late-phase images, the average was 72 HU (±13.4) and the measurements ranged from 68 HU (±14) in the no enhancement (C_NE_) group to 78 HU (±15) in the early enhancement without washout (C_NW_) group.

The logistic regression analysis for the TICI III scores, with the early enhancement without washout group (C_NW_) as the reference, showed an OR of 1.82 (CI: 0.57 to 5.66, *p* = 0.30) for no enhancement (C_NE_), an OR of 1.69 (CI: 0.51 to 5.63, *p* = 0.39) for early enhancement with washout (C_W_), and an OR of 4.04 (CI: 0.97 to 21.02, *p* = 0.067) for late enhancement (C_LE_). The results remained unchanged when adjusting for clot length in the analysis. To compare, the logistic regression for the TICI III scores using standard perviousness (SP) without dynamic groups showed no association (OR of 1.00 for CTA, CI: 0.98 to 1.03, *p* = 0.96; OR of 1.02 for CTV, CI: 0.98 to 1.07, *p* = 0.43).

The linear regression analysis for the NIHSS scores at discharge, with the C_NW_ group as the reference and adjusting for the NIHSS score at admission, also did not show any significant differences. The linear regression analysis for the NIHSS scores at discharge (C_NW_ group as reference) resulted in beta coefficients of −1.24 (CI: −4.66 to 2.18, *p* = 0.47), −1.17 (CI: −4.70 to 2.37, *p* = 0.51), and −3.05 (CI: −6.74 to 0.64, *p* = 0.10) for C_NE_, C_W_, and C_LE_, respectively. The beta coefficients for the mRS scores at 3 months (adjusting for the mRS score at admission) were −0.15 (CI: −1.53 to 1.24, *p* = 0.83), −0.85 (CI: −2.31 to 0.60, *p* = 0.25), and −1.30 (CI: −2.84 to 0.24, *p* = 0.097) for C_NE_, C_W_, and C_LE_, respectively.

Out of the 137 patients, 10 did not require an intervention, as the thrombus was not visible or was too distal by the time the first interventional angiography was produced. In the remaining 127 patients, the Poisson regression model showed a ratio of 0.76 (CI: 0.56 to 1.03, *p* = 0.077) for no enhancement, 0.79 (CI: 0.57 to 1.09, *p* = 0.15) for early enhancement with washout, and 0.73 (CI: 0.51 to 1.03, *p* = 0.076) for late enhancement.

## 4. Discussion

In this paper, we propose the novel imaging biomarker of dynamic perviousness of occlusive thrombus to refine the concept of the contrast material–clot interaction in a time-dependent way, from the early arterial up to the late venous phase. Based on the uptake curve, we were able to distinguish four different categories of contrast–clot interaction, namely no enhancement, late enhancement, and early enhancements with and without washout. Our data show that where standard (non-dynamic) perviousness had no predictive value for the outcomes, dynamic perviousness was able to provide additional information on the thrombus characteristics during MT; late-enhancing thrombi had the highest odds for successful revascularization, whereas early enhancement without washout tended to correlate with unfavorable results.

The value of standard, non-dynamic perviousness is debated due to the contradictory data. An early study by Santos et al. [4] in 184 patients from the Multicenter Randomized Clinical trial of Endovascular treatment of acute ischemic stroke in the Netherlands (MR CLEAN) showed that perviousness is associated with an improved functional outcome and recanalization. However, research by Byun et al. later could not detect a significant difference in rates of first-pass recanalization or overall revascularization success using stent retrievers in a set of 52 patients. Another study by Santos et al. [13] examined the effect of dedicated multiphase CTA with two additional acquisitions 8 and 16 s after the initial arterial phase and found no additional value for outcome prediction. A meta-analysis with pooled data from seven studies, including 443 patients, suggested that with increasing perviousness, the additional benefits of mechanical thrombectomy diminish [9]. A study of 75 patients using data from M1 occlusions found that non-enhanced CT could predict a cardioembolic cause but did not include any dynamic data from late-phase CE CT [14]. In 2023, Pilato et al. studied 100 patients and found that reduced perviousness (in a single contrast phase) was associated with less distal embolization in M1 occlusions [15]. A voxel-wise characterization of 3D thrombi performed by our group showed dynamic perviousness to be a better predictor of MT outcomes compared with standard perviousness [16].

Under the assumption that the available research did not fully capture the dynamic enhancement pattern due to the absence of late-phase imaging or a short wait between the early arterial and later phases of no more than 16 s, we used CE CT data from instances where a late venous phase had additionally been performed after a median of 43 s. We hypothesized that the clot attenuation would follow discrete patterns, depending on the density and permeability, and split the sample into four separate enhancement patterns. There were observable tendencies that may be of interest, whereby the technical and clinical outcomes in patients with late enhancement thrombi seemed to be better than with no enhancement, with a higher change for successful intervention and lower scores for the NIHSS and mRS. The patients with early enhancement without washout tended to require more passes to achieve recanalization. We hypothesize that this may be due to the underlying histological structure of the clot; thrombi without early washout may have higher tissue densities, allowing longer accumulation of the contrast agent in the thrombus than in thrombi with relatively low tissue densities, or it may relate to the degree of occlusion and collateral blood flow, whereby a clot that shows no washout may not receive as much fresh blood as one that shows washout. On the other hand, the dynamic contrast uptake characteristics may be also influenced by the geometric properties of the thrombus, allowing various degrees of contrast penetration. We surmise that some of these effects may not have been observed so far due to the fact that a late venous phase was not taken into account in previous studies.

There are several limitations to this study. First, due to the retrospective nature of the design, only the patients who had full imaging data of sufficient quality available at our institution could be included, which may have led to bias. Second, due to its retrospective design, we did not used a fixed time delay between the NCCT, CTA, and CTV acquisitions. This may have introduced a potential bias into the TAI computation results. Third, the imaging resolution may have limited the reliability of density measurements, as in similar previous studies. Fourth, the relatively low sample size with a high revascularization rate may have prevented this study from reaching significance.

Overall, further studies are needed to understand whether late-phase imaging with dynamic enhancement of the thrombus has additional value for outcome prediction or treatment planning. Radiological–pathological correlations of the uptake types may explain some of the observed effects, and we are planning to perform a study on histological slides of extracted thrombi.

## 5. Conclusions

Here, a dynamic perviousness assessment had higher predictive value as compared to standard perviousness in this study. Late enhancement patterns of thrombi had four times higher odds for successful revascularization as compared to clots with early enhancement without washout. An assessment of dynamic clot perviousness in large vessel occlusions, including late-phase CTV imaging, may have more important potential to predict successful recanalization and clinical outcomes after mechanical thrombectomy.

## Figures and Tables

**Table 1:** Demographic data, stroke characteristics, and treatment outcomes for the study population. ICA = internal carotid artery; MCA = middle cerebral artery; NIHSS = National Institutes of Health Stroke Scale; TICI = Thrombolysis in Cerebral Infarction.

Characteristic	Value
Median age (Range)	75.5 (17–94)
Female *N* (%)	56 (41)
Stroke severity at presentation *N* (%)	
Minor (NIHSS 0–4)	14 (10.2)
Moderate (NIHSS 5–15)	57 (41.6)
Moderate to severe (NIHSS 16–20)	41 (29.9)
Severe (NIHSS 20+)	25 (18.2)
Occlusion location *N* (%)	
ICA intracranial	43 (31.4)
MCA M1	69 (50.3)
MCA M2	19 (13.9)
Basilar artery	6 (4.4)
Average clot length mm (SD)	16 (10)
TICI *N* (%)	
0-2b	28 (20.4)
2c-3	109 (79.6)

**Table 2:** Imaging data including clot characteristics and density values, as well as the recanalization success, required passes, and clinical outcome at 3 months, depending on enhancement type. C_NE_ = no enhancement; C_NW_ = early enhancement, no washout; C_W_ = early enhancement with washout; C_LE_ = late enhancement; NIHSS = National Institutes of Health Stroke Scale; mRS = Modified Rankin Scale.

	Overall	C_NE_	C_NW_	C_W_	C_LE_
***N***	137	49	22	37	29
Age mean (SD)	72.9 (14.2)	70.2 (15.9)	78 (12.3)	71.5 (13.8)	75.5 (12.1)
Clot length mean (SD)	18.2 (10.4)	18.9 (9.4)	20.2 (11.4)	14.7 (8.1)	19.9 (13)
NCCT mean HU (SD)	64.4 (9.6)	68.6 (8.1)	62.5 (9.6)	61.3 (10.8)	62.5 (7.9)
CTA mean HU (SD)	74.4 (22)	67 (8.7)	73.1 (9.8)	95.3 (31.1)	61.4 (8.1)
CTV mean HU (SD)	71.2 (13.4)	68.7 (8.2)	78.2 (14.6)	68.5 (13.4)	79.8 (16.6)
NIHSS mean (SD)	13.7 (6.6)	13.5 (6.9)	17 (6.9)	12.2 (6.3)	13.6 (5.5)
Recanalization success *N* (%)	109 (79.6)	39 (79.6)	15 (31.7)	29 (78.4)	26 (89.6)
Number of passes mean (SD)	2.4 (2.0)	2.5 (2.2)	2.7 (2.1)	2.0 (1.7)	2.6 (1.9)
mRS at 3 months mean (SD)	3.3 (2.3)	3.7 (2.2)	3.9 (2.4)	2.9 (2.2)	2.7 (2.4)

## Data Availability

Data are available upon reasonable request.
